# Research progress of polyunsaturated fatty acids in inflammatory bowel disease: from mechanisms to treatment

**DOI:** 10.3389/fphar.2026.1971572

**Published:** 2026-10-14

**Authors:** Jian-long Zhang, Ying Liu, Jia Liu, Wan-neng Yan, Ze Yu

**Affiliations:** 1 The Laboratory of Cytobiology and Molecular Biology, Zhoushan Hospital, Wenzhou Medical University, Zhoushan, Zhejiang, China; 2 Department of Pharmacy, Zhoushan Hospital, Wenzhou Medical University, Zhoushan, Zhejiang, China; 3 School of Agriculture and Biotechnology, Sun Yat-sen University, Shenzhen, Guangdong, China; 4 Shenzhen Zhongjia Bio-medical Technology Co., LTD, Shenzhen, China; 5 Department of Proctology, Zhoushan Hospital, Wenzhou Medical University, Zhoushan, Zhejiang, China; 6 Department of Surgery, Zhoushan Hospital, Wenzhou Medical University, Zhoushan, Zhejiang, China

**Keywords:** gut microbiota, inflammatory bowel disease (IBD), mechanism, polyunsaturated fatty acids, treatment

## Abstract

Inflammatory bowel disease (IBD) imposes a growing clinical burden through recurrent intestinal inflammation, malnutrition, treatment-related toxicity, and incomplete mucosal healing. Polyunsaturated fatty acids (PUFAs) are plausible dietary regulators of this burden, yet their effects cannot be reduced to a simple anti-inflammatory role for n-3 PUFAs and a pro-inflammatory role for n-6 PUFAs. The proposed pre-clinical concept of PUFA-induced metabolic enteritis links dietary lipid exposure to epithelial stress, lipid peroxidation, and intestinal inflammation in genetically or metabolically susceptible pre-clinical models. Its human counterpart, however, remains incompletely defined. This narrative review integrates evidence across PUFA-derived lipid mediators, the gut microbiota, intestinal barrier function, mucosal immunity, and the glutathione peroxidase 4 (GPX4)-ferroptosis axis. We distinguish observational associations from interventional evidence and examine why clinical trials of eicosapentaenoic acid and docosahexaenoic acid have produced inconsistent results. We also evaluate dietary patterns, PUFA supplements, microbiome-directed approaches, specialized pro-resolving mediators, oxylipin pathways, and soluble epoxide hydrolase inhibition. Current evidence remains pre-clinical. Our review provides an approach and insights for managing IBD before PUFA-directed interventions are incorporated into precision treatment for IBD.

## 1 Introduction

Inflammatory bowel disease (IBD), comprising Crohn’s disease (CD) and ulcerative colitis (UC), is a chronic, relapsing group of immune-mediated intestinal disorders. Global estimates indicate that millions of people live with IBD, while incidence continues to rise in many industrializing regions (Ruan et al., 2025). CD can affect any gastrointestinal segment and often follows a progressive, structurally damaging course (Roda et al., 2020). UC is restricted to the colon but still carries substantial risks of hospitalization, colectomy, and colorectal cancer (Gros and Kaplan, 2023). Despite major advances in biologics and small molecules, therapeutic failure, loss of response, infection risk, and unequal access remain clinically important.

Nutrition is relevant to IBD for two distinct reasons. First, inflammation, malabsorption, gastrointestinal loss, reduced intake, and restrictive diets create a substantial risk of malnutrition. Contemporary guidance therefore recommends systematic nutritional screening and individualized support throughout the disease course (Bischoff et al., 2023). Second, diet is an environmental exposure that can modify microbial metabolism, epithelial stress, and mucosal immunity. Malnutrition and inflammation can reinforce one another, creating a cycle that impairs recovery and treatment response (Massironi et al., 2023). These observations have intensified interest in dietary factors that might complement, but not replace, evidence-based medical therapy.

Polyunsaturated fatty acids (PUFAs) are central to this debate. Linoleic acid (LA) and alpha-linolenic acid (ALA) are essential dietary fatty acids and precursors of longer-chain n-6 and n-3 PUFAs. Their derivatives participate in membrane organization, energy metabolism, immune signalling, inflammation, and tissue repair. However, a nutrient-centered model that labels all n-6 PUFAs as harmful and all n-3 PUFAs as protective is biologically incomplete. PUFA effects depend on molecular species, dose, tissue incorporation, enzymatic processing, oxidative environment, and host susceptibility.

The proposed pre-clinical concept of PUFA-induced metabolic enteritis provides a useful mechanistic framework but also creates a terminology problem. Metabolic enteritis describes intestinal inflammation driven by nutrient-induced metabolic stress, particularly in experimental models with impaired epithelial stress responses or antioxidant defence (Adolph et al., 2022). A pivotal translational study connected PUFA exposure with epithelial stress signatures and disease activity in CD cohorts (Schwärzler et al., 2022). Earlier work showed that a PUFA-enriched Western diet provoked Crohn-like small-intestinal inflammation in mice with reduced epithelial GPX4 activity (Mayr et al., 2020). These studies do not establish metabolic enteritis as a clinical diagnosis in humans. Rather, they identify a mechanistic endotype that may aggravate disease in susceptible patients.

This review examines how PUFAs regulate intestinal inflammation through coupled microbial, epithelial, immune, and lipid-mediator networks. We critically compare clinical and pre-clinical evidence, highlight contradictory findings, and identify specific translational priorities.

## 2 Definition of dietary PUFAs

Humans cannot introduce double bonds at the n-3 or n-6 positions and must obtain LA and ALA from food. LA can be elongated and desaturated to arachidonic acid (AA), while ALA can be converted inefficiently to eicosapentaenoic acid (EPA) and docosahexaenoic acid (DHA). Marine foods provide preformed EPA and DHA, whereas seed oils, nuts, and many processed foods provide variable amounts of LA and ALA. Dietary intake does not map directly onto tissue function because absorption, beta-oxidation, membrane incorporation, and enzymatic conversion vary among individuals.

PUFA-derived mediators form a network rather than two opposing linear pathways. Cyclooxygenases, lipoxygenases, and cytochrome P450 enzymes convert PUFAs into prostanoids, leukotrienes, hydroxy-fatty acids, epoxy-fatty acids, resolvins, protectins, maresins, and related oxylipins (Christie and Harwood, 2020). AA-derived prostaglandins and leukotrienes can amplify inflammation, yet some AA-derived lipoxins and epoxy-eicosatrienoic acids have anti-inflammatory or pro-resolving actions. EPA and DHA can compete with AA for membrane incorporation and enzymatic metabolism. They also generate specialized pro-resolving mediators (SPMs), but their availability does not guarantee efficient SPM biosynthesis. Thus, the balance among substrates, enzymes, receptors, and degradation pathways is more informative than a dietary n-6/n-3 ratio alone.

The Western dietary pattern has been linked to a greater risk of developing CD in some prospective studies, although findings are not consistent across cohorts (Meyer et al., 2025) (Figure 1). n-3 PUFAs influence inflammation through both mediator-dependent and mediator-independent mechanisms. They can alter membrane microdomains, regulate nuclear receptors, reduce some nuclear factor kappa B-dependent responses, and provide substrates for pro-resolving mediators (Calder, 2020). Nevertheless, their multiple double bonds also make them susceptible to peroxidation. When antioxidant defence is impaired, EPA and DHA can contribute to lipid-peroxide burden. This conditional vulnerability helps reconcile their generally pro-resolving biology with the adverse response observed in selected GPX4-restricted models.

**FIGURE 1:**
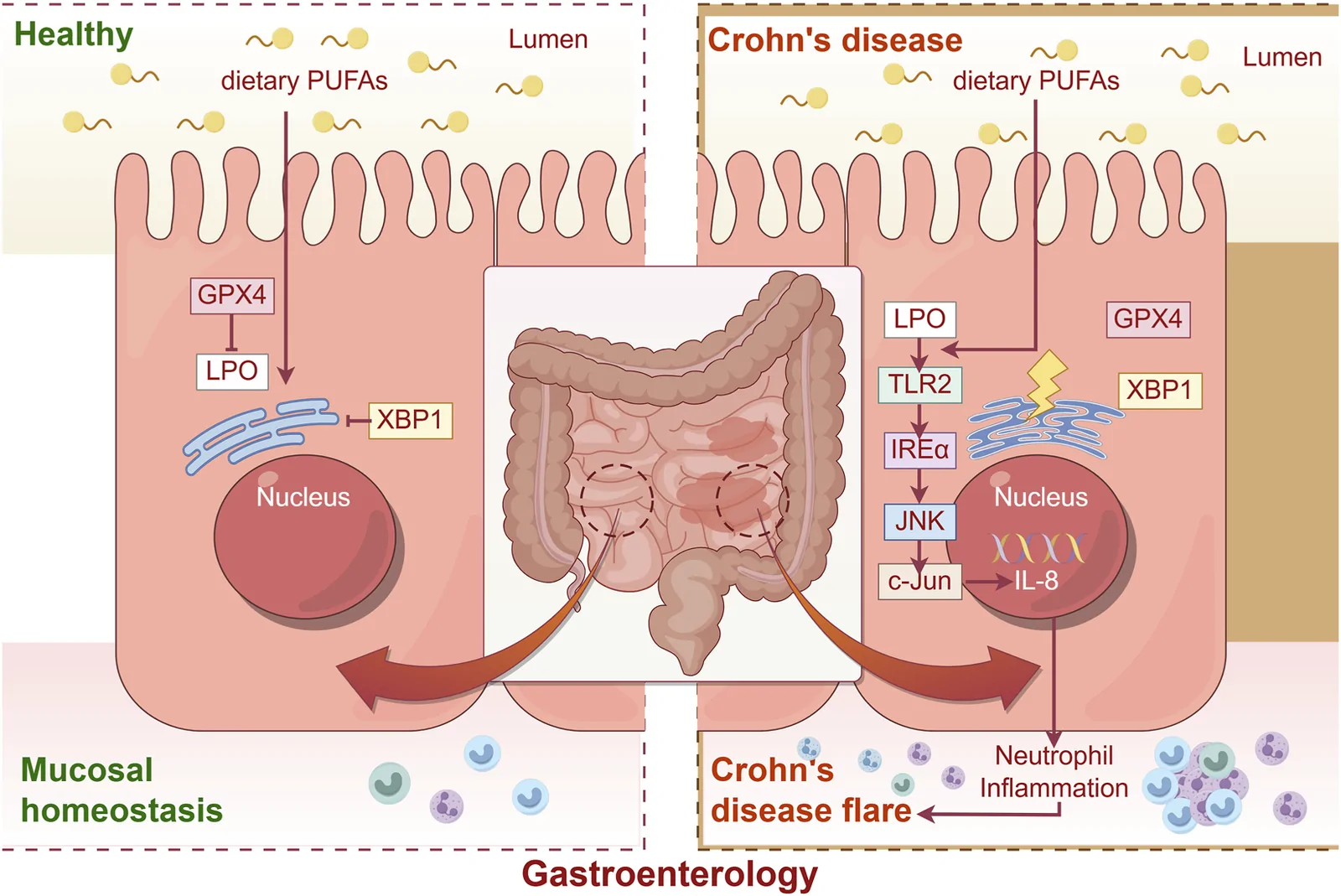
Proposed model of PUFA-associated intestinal inflammation based on pre-clinical and translational studie. This schematic integrates findings from specific mouse models and translational experiments reported by Schwärzler et al. (2022) and Mayr et al. (2020). The left panel illustrates epithelial homeostasis, in which GPX4 limits lipid peroxidation (LPO) and XBP1 supports endoplasmic reticulum (ER) homeostasis. The right panel summarizes experimental PUFA responses in susceptible epithelial settings, including intestinal epithelial Xbp1-deficient and Gpx4-haploinsufficient mice. In these models, PUFA exposure promotes LPO and ER stress, with TLR2-dependent IRE1α–JNK–c-Jun signaling implicated in chemokine production and intestinal inflammation.

## 3 Mechanistic network linking PUFAs to intestinal inflammation

### 3.1 Gut microbiota and microbial metabolism

IBD-associated dysbiosis is characterized more consistently by functional instability than by a single disease-specific taxonomic signature. Longitudinal multi-omics profiling has revealed increased facultative anaerobes, reduced obligate anaerobes, altered bile-acid pools, and disrupted short-chain fatty acid (SCFA) metabolism during active disease (Lloyd-Price et al., 2019). Independent metagenomic and metabolomic work has likewise identified disease-associated microbial functions and metabolite profiles that outperform taxonomic signals alone (Franzosa et al., 2019). These findings caution against assigning fixed beneficial or harmful labels to individual genera. SCFAs, especially butyrate, acetate, and propionate, connect microbial fermentation to epithelial energy supply, tight-junction regulation, and immune tolerance. A recent quantitative synthesis reported lower faecal SCFA concentrations across IBD subgroups, with pronounced reductions during active disease (Chulenbayeva et al., 2025). However, faecal concentration reflects production, absorption, transit, and sampling conditions. It should not be treated as a direct measure of mucosal exposure.

n-3 PUFA supplementation can modify the human microbiota, but the effect is modest and heterogeneous. In a randomized crossover study of healthy adults, EPA/DHA increased several SCFA-producing taxa without changing overall alpha or beta diversity (Watson et al., 2018). The population was small, healthy, and exposed to a high supplemental dose, which limits extrapolation to active IBD. In contrast, krill oil altered microbial and metabolite profiles and attenuated inflammation in cell, pig, and mouse models (Liu et al., 2020). Krill oil also contains phospholipids and astaxanthin, so its effects cannot be attributed solely to EPA and DHA. PUFAs may influence microorganisms through changes in luminal substrates, bile acids, oxygen tension, antimicrobial responses, and epithelial secretions. Microbes can reciprocally transform dietary lipids and alter host oxylipin production. This bidirectional relationship makes microbiota composition both a mediator and a potential effect modifier. Antibiotics, biologics, dietary fibre, and disease activity can all confound the observed association between PUFA exposure and microbial structure.

### 3.2 Intestinal barrier integrity

The intestinal barrier comprises epithelial cells, mucus, antimicrobial peptides, tight and adherens junctions, immune cells, and the vascular interface. Inflammation can disrupt this system and increase the access of microbial products to the lamina propria. n-3 PUFAs have been reported to support tight-junction organization, epithelial repair, and mucosal homeostasis, but much of the evidence comes from cell culture and animal studies (Wawrzyniak et al., 2021). Human data are more limited. An exploratory analysis of the randomized LIBRE trial associated higher plasma DHA with lower lipopolysaccharide-binding protein and faecal zonulin during a Mediterranean dietary intervention (Seethaler et al., 2023). The analysis involved women at elevated breast-cancer risk rather than patients with IBD. Zonulin assays also have recognized specificity limitations. Therefore, the study supports biological plausibility but does not establish clinical efficacy in IBD.

Barrier effects may be mediated indirectly through SPMs, SCFAs, immune-cell polarization, or reduced inflammatory signalling (Durkin et al., 2021). Conversely, excessive peroxidizable lipid exposure can increase epithelial stress when GPX4 activity is insufficient. The same PUFA can therefore support barrier repair in one context and aggravate epithelial injury in another.

### 3.3 Lipid mediators and active resolution

Resolution is an active biological programme rather than passive disappearance of inflammation. In human IBD biopsies, n-3 docosapentaenoic acid-derived protectin D1 and resolvin D5 pathways are detectable, and both mediators reduced leukocyte recruitment and experimental intestinal injury (Gobbetti et al., 2017). Maresin 2 promoted epithelial migration and mucosal repair in experimental colitis and human epithelial systems (Miranda et al., 2023). Multiple SPMs also enhanced wound repair in Caco-2 cultures through G-protein-coupled receptor and mitogen-activated protein kinase signalling (Storniolo et al., 2022). These results identify repair mechanisms, but they do not demonstrate that oral fish oil produces therapeutic tissue concentrations of specific SPMs.

Recent syntheses position SPMs at the interface of barrier repair, immune reprogramming, and microbial ecology (Albuquerque-Souza and Dalli, 2025). Translational barriers remain substantial. SPMs are chemically labile, occur at low concentrations, and require rigorously validated mass-spectrometric methods. Receptor selectivity, pharmacokinetics, formulation, and disease-stage specificity also require clarification.

### 3.4 Oxylipin imbalance and the sEH pathway

Oxylipin profiles integrate substrate availability with cyclooxygenase, lipoxygenase, cytochrome P450, and hydrolase activities. A case-control study reported higher prostaglandins and leukotrienes, lower n-3 PUFAs and 18-hydroxyeicosapentaenoic acid, and altered pro-resolving pathways in CD and UC (Ben-Mustapha et al., 2024). The cohort was small and cross-sectional, so the observed profile might reflect inflammation, treatment, or altered intake rather than cause disease. Earlier mucosal lipidomics in treatment-naive UC similarly showed increased n-6-related oxylipins and lower n-3-related endocannabinoids (Diab et al., 2019). These studies support pathway dysregulation but not a universal diagnostic signature.

Soluble epoxide hydrolase (sEH), encoded by EPHX2, converts epoxy-fatty acids into generally less active diols. This reaction can reduce the persistence of anti-inflammatory epoxy-eicosatrienoic acids and related mediators (Ren et al., 2024). Genetic sEH deficiency attenuated dextran sodium sulfate-induced colitis and colitis-associated carcinogenesis in mice (Zhang et al., 2013). Pharmacological work in gastrointestinal injury models also suggests that sEH inhibition shifts the oxylipin pool toward epoxy-fatty acids and strengthens epithelial defence (Jones et al., 2019). Nevertheless, no sEH inhibitor is currently established as an IBD therapy. The available evidence remains pre-clinical, and systemic vascular or metabolic effects will require careful safety evaluation.

### 3.5 The GPX4-lipid peroxidation-ferroptosis axis

PUFAs are essential structural lipids but also preferred substrates for lipid peroxidation. Acyl-CoA synthetase long-chain family member 4 (ACSL4) enriches membranes with long-chain PUFAs and increases ferroptosis sensitivity (Doll et al., 2017). GPX4 uses glutathione to reduce phospholipid hydroperoxides and thereby restrains ferroptosis, an iron-dependent form of regulated cell death. Gastrointestinal ferroptosis is additionally modified by iron handling, system Xc-, ferroptosis suppressor protein 1, mitochondrial metabolism, and cell-specific antioxidant capacity (Escuder-Rodríguez et al., 2024). In GPX4-restricted intestinal epithelial cells, PUFA exposure increases lipid peroxidation and inflammatory cytokine production. Importantly, epithelial cell death was not required for the inflammatory phenotype in the original mouse model (Mayr et al., 2020). Pre-clinical PUFA-induced metabolic enteritis should therefore not be described as synonymous with ferroptosis. However, a 2024 study reported that regulatory T (Treg) cells preferentially incorporated PUFAs into membrane phospholipids during high-fat feeding, which increased their susceptibility to ferroptosis and aggravated experimental colitis (Yan et al., 2024). This mechanism links nutrient exposure to loss of immune tolerance. However, the dietary model used high-fat exposure, and direct evidence in human intestinal Treg cells remains unavailable. The integrated pro-inflammatory network and its principal evidence boundaries are summarized in Figure 2.

**FIGURE 2:**
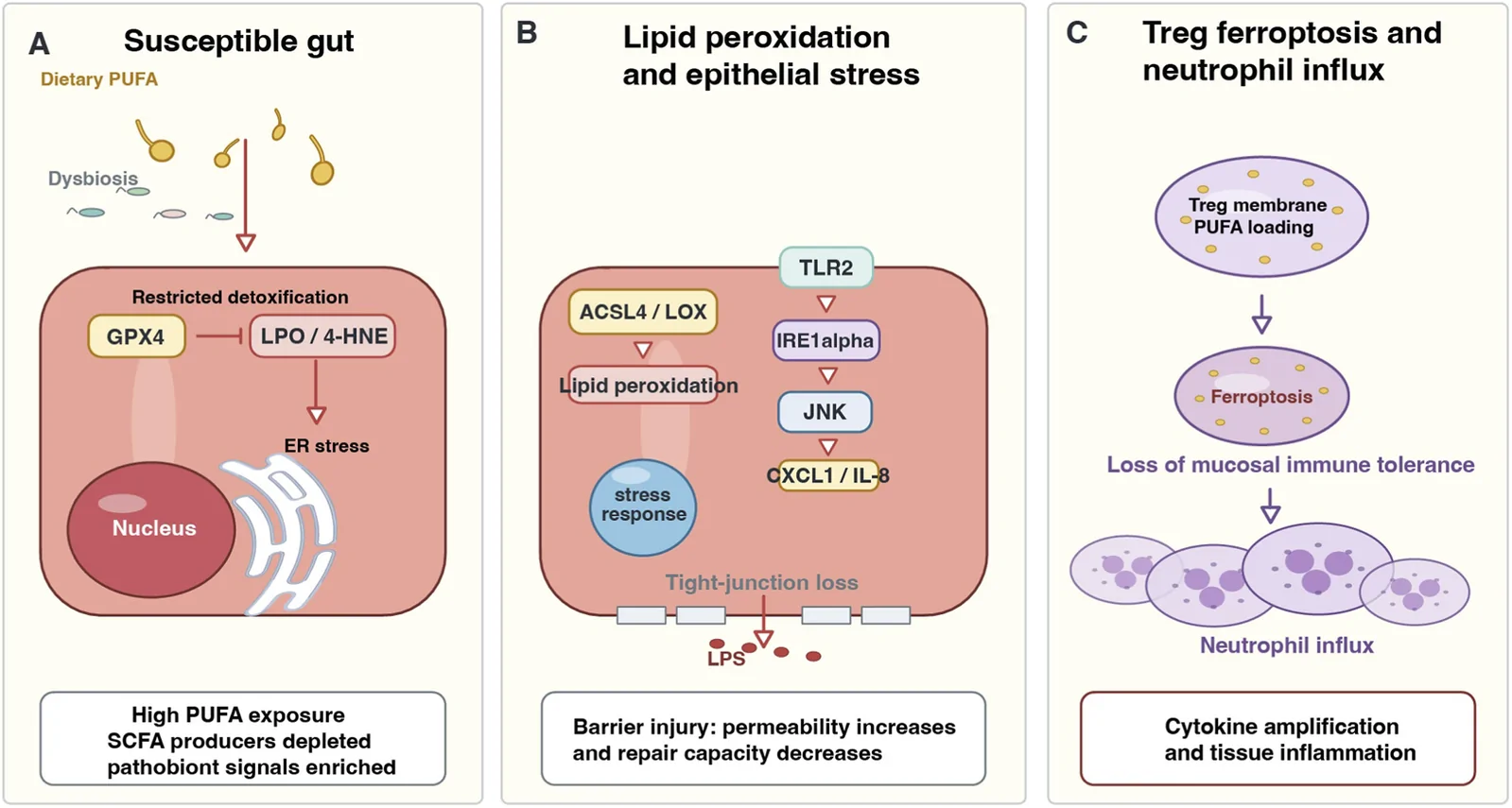
Mechanistic network of pre-clinical PUFA‐induced metabolic enteritis in a susceptible gut **(A)** Excess dietary PUFA exposure can generate oxidized epitopes and epithelial endoplasmic-reticulum stress. When glutathione peroxidase 4 (GPX4)‐dependent lipid-peroxide detoxification is restricted, lipid peroxidation and **(B)** TLR2‐IRE1alpha‐JNK signalling increase chemokine production and neutrophil recruitment. **(C)** Regulatory T (Treg) cells can also become vulnerable to ferroptosis after excessive membrane PUFA loading. In parallel, microbial dysbiosis, reduced short-chain fatty acid (SCFA) output, tight‐junction disruption, and translocation of lipopolysaccharide (LPS) or other microbial products amplify inflammation. The resulting feed‐forward loop further impairs microbial ecology, redox control, and barrier repair. The phenotype is established mainly in pre-clinical models and should not be equated with human IBD.

### 3.6 Cross-talk among microbiota, barrier, and immunity

These mechanisms operate as a coupled system. PUFA-driven epithelial stress can alter antimicrobial and chemokine outputs, reshaping microbial niches and recruiting neutrophils. Dysbiosis can reduce SCFA production, weaken epithelial energy supply, and change bile-acid and tryptophan metabolites. Barrier injury then increases the delivery of lipopolysaccharide and other microbial signals to immune cells. Activated leukocytes generate reactive species and cytokines that further impair tight junctions and antioxidant defence.

This network also explains why isolated endpoints can mislead. A supplement may increase a nominally beneficial taxon without improving mucosal inflammation. Conversely, a clinical response may occur without a large change in community diversity because microbial transcription or metabolite output has shifted. Mechanistic studies should therefore measure microbial function, barrier integrity, lipid mediators, immune state, and disease activity in the same longitudinal samples.

## 4 Human and pre-clinical evidence

### 4.1 Observational and epidemiological evidence

Prospective epidemiology provides evidence of association, not causation. In the European Prospective Investigation into Cancer and Nutrition cohort, higher LA intake was associated with incident UC (IBD in EPIC Study Investigators et al., 2009). However, only 126 cases were identified, exposure relied on food-frequency questionnaires, and residual confounding remained possible. In two large US cohorts, cumulative total fat and overall n-3 or n-6 PUFA intake were not associated with CD or UC, although specific long-chain fatty acids showed divergent signals (Ananthakrishnan et al., 2014). Differences in population, dietary pattern, repeated exposure assessment, and fatty-acid specification may explain the inconsistency.

Dietary inflammatory potential was associated with incident CD, but not UC, across three large prospective cohorts (Lo et al., 2020). Meta-analysis has linked fish intake and long-chain n-3 PUFA exposure with lower risk of selected IBD outcomes, while total n-3 intake showed no consistent association (Mozaffari et al., 2020). Another dose-response meta-analysis found no robust association between overall PUFA intake and UC after accounting for study heterogeneity (Wang et al., 2017). A 2025 synthesis of prospective cohorts associated inflammatory and ultra-processed dietary patterns with higher CD risk, while fibre-rich and Mediterranean patterns were associated with lower risk (Meyer et al., 2025). These pattern-level findings should not be attributed to PUFAs alone. A separate systematic review concluded that evidence linking dietary inflammatory potential to IBD progression remained heterogeneous (Anneberg et al., 2025). Major sources of bias include recall error, changing diet after preclinical symptoms, total-energy misreporting, supplement use, smoking, disease location, and socioeconomic factors. Observational studies rarely capture chemical form, oxidation status, or food matrix. Most also cannot distinguish whether circulating PUFA depletion reflects low intake, inflammation-driven consumption, malabsorption, or treatment.

### 4.2 Clinical supplementation trials

A systematic review and meta-analysis of randomized trials found little or no reliable effect of long-term n-3, n-6, or total PUFA modification on IBD outcomes or inflammatory markers (Ajabnoor et al., 2021). The two large EPIC trials randomized 738 patients with quiescent CD to 4 g/day omega-3 free fatty acids or placebo (Table 1). Relapse was not significantly reduced in either trial (Feagan et al., 2008). Earlier systematic review evidence likewise did not support routine omega-3 supplementation for active or inactive IBD (Cabré et al., 2012).

**TABLE 1:** Clinical evidence on omega-3 supplementation in inflammatory bowel disease.

Study	Population	Formulation	Daily dose	Duration	Outcome
EPIC-1 Double-blind RCT	Adults with CD; Remission for 3–12 months; CDAI<150. n = 363	Delayed-release omega-3 free fatty acid capsules, MCT placebo	4 g study product; EPA 50%–60%, DHA 15%–25%	52 weeks	1-year relapse: 31.6% vs. 35.7% (placebo); P = 0.30
EPIC-2 Double-blind RCT	Adults with CD; Steroid-induced remission; CDAI<150. n = 375; steroids tapered after randomization	Same formulation and comparator as EPIC-1	Same target dose as EPIC-1	58 weeks	1-year relapse: 47.8% vs. 48.8% (placebo); P = 0.48
Meta-Analysis	83 RCTs overall (Participants = 41,751); 13 recruited existing IBD (7 UC, 6 CD)	Long-chain n-3 interventions, mainly fish-oil supplements; comparators varied. Review also assessed other PUFAs	Existing-IBD trials: EPA 1.12–4.5 g and DHA 0.73–2.4 g	≥24 weeks eligible; existing-IBD trials: 6–24 months	IBD relapse: RR 0.85 (95% CI 0.72–1.01); 10 trials, 1,196 participants; low-certainty evidence
Review	19 RCTs; active UC(n = 7), inactive UC(n = 4), active CD(n = 2), inactive CD(n = 6); Adults and children	Fish oil, free fatty acids, ethyl esters, and enriched nutritional formulas; comparators varied	EPA 1.8 g + DHA 0.9 g (Belluzzi 1996); EPA 3.3 g + DHA 1.8 g (Lorenz-Meyer 1996)	Active UC: 2–6-month treatment periods; active CD: 6–9 weeks; maintenance: approximately 1–2 years	No consistent maintenance benefit

Abbreviations: CD, Crohn’s disease; UC, ulcerative colitis; IBD, inflammatory bowel disease; CDAI, Crohn’s Disease Activity Index; RCT, randomized controlled trial; EPA, eicosapentaenoic acid; DHA, docosahexaenoic acid; MCT, medium-chain triglycerides; RR, risk ratio; CI, confidence interval; PUFA, polyunsaturated fatty acid.

Several factors may obscure a true subgroup effect. Trials used different ethyl-ester, triglyceride, free-fatty-acid, or food-based preparations. Dose, EPA/DHA ratio, oxidation stability, adherence, background diet, and treatment duration varied. Some studies enrolled active disease, whereas others targeted maintenance. Baseline fatty-acid status, GPX4 activity, disease location, microbiota, and concomitant medication were seldom used for stratification. Placebo oils may also have biological activity. These design differences prevent a simple conclusion that n-3 PUFAs are universally ineffective or universally beneficial.

### 4.3 Diet patterns and food-based interventions

Natural food intake and purified supplements are not equivalent exposures. Foods deliver PUFAs within protein, fibre, antioxidants, micronutrients, and complex lipid structures. A randomized trial in adults with mild-to-moderate CD found similar symptomatic remission with the specific-carbohydrate and Mediterranean diets, while objective biomarker responses were uncommon (Lewis et al., 2021). In quiescent UC, a small randomized trial found lower faecal calprotectin and higher SCFAs with a Mediterranean pattern than with a habitual Canadian diet (Haskey et al., 2023). The sample size was small, and the intervention changed many dietary components simultaneously.

The Crohn’s disease exclusion diet has shown feasibility and clinical activity in open-label randomized studies (Yanai et al., 2022). Multi-omics analysis of newly diagnosed CD has also associated Mediterranean-diet adherence with microbial and metabolic features linked to disease course (Godny et al., 2025). A pilot randomized trial of a modified anti-inflammatory diet reported high adherence and exploratory improvements in clinical measures (Marsh et al., 2024). None of these interventions isolates a PUFA effect. They instead support the clinical relevance of food matrices and whole-diet patterns (Table 2).

**TABLE 2:** Comprehensive progress in human research.

Evidence	Representative finding	Principal limitation
Prospective n-6 exposure	Higher linoleic acid intake was associated with incident UC in EPIC (Yan et al., 2024)	Few cases, food-frequency questionnaires, and residual confounding.
Repeated cohort assessment	Total n-3 and n-6 intake was not associated with CD or UC in two US cohorts (IBD in EPIC Study Investigators et al., 2009)	Predominantly female health professionals and limited chemical-form data.
Dietary pattern epidemiology	Inflammatory and ultra-processed patterns were linked to higher CD risk (Ananthakrishnan et al., 2014; Wang et al., 2017; Anneberg et al., 2025)	Observational associations cannot establish causality or isolate PUFAs.
Fish and n-3 meta-analysis	Fish or long-chain n-3 exposure showed selected protective associations (Lo et al., 2020; Mozaffari et al., 2020)	Heterogeneous case-control and cohort methods; inconsistent total n-3 findings.
EPA/DHA supplementation	Long-term trials and the EPIC studies did not consistently prevent relapse (Ajabnoor et al., 2021; Feagan et al., 2008; Cabré et al., 2012)	Formulation, dose, disease stage, placebo oil, adherence, and background diet varied.
Whole-diet intervention	Mediterranean, specific-carbohydrate, CDED, and anti-inflammatory diets showed mixed clinical and biomarker effects (Lewis et al., 2021; Haskey et al., 2023; Yanai et al., 2022; Godny et al., 2025; Marsh et al., 2024)	Small or open-label trials changed many nutrients simultaneously.

### 4.4 Evidence from microbiome-directed studies

Dietary interventions do not consistently generate a reproducible IBD microbiome signature. A systematic review of intervention studies found substantial heterogeneity in diets, sequencing methods, sampling time points, and clinical endpoints (Nieva et al., 2024). Many studies reported no significant shift in diversity or disease activity. In healthy adults, 500 mg/day omega-3 supplementation changed selected taxa and branched SCFAs, but inulin produced larger microbial effects (Vijay et al., 2021). These findings do not establish that combined probiotics, prebiotics, and PUFAs improve IBD.

Evidence for synergy between probiotics or prebiotics and PUFAs remains predominantly pre-clinical or extrapolated from non-IBD populations. Future trials should predefine strain identity, dose, viability, fibre substrate, PUFA formulation, and disease phenotype. They should also separate symptom improvement from objective mucosal response.

## 5 Therapeutic strategies and translational barriers

### 5.1 Food-based intake versus PUFA supplementation

Clinical counselling should distinguish adequate dietary intake from high-dose supplementation. A balanced dietary pattern can improve overall nutrient density and reduce ultra-processed food exposure without assuming that a specific n-6/n-3 ratio is therapeutic. Purified EPA/DHA supplements produce larger and more controllable changes in circulating fatty acids, but they also introduce issues of oxidation, gastrointestinal tolerance, dose, and formulation. Routine high-dose omega-3 supplementation cannot currently be recommended to induce or maintain IBD remission. This conclusion does not preclude correcting inadequate intake or using nutrition support for malnutrition. It instead reflects the absence of consistent disease-modifying benefit. Trials should report background diet, supplement composition, oxidation indices, adherence, red-blood-cell fatty acids, and concomitant drugs.

### 5.2 Lipid-mediator pharmacology

Targeting downstream lipid mediators may be more precise than increasing precursor intake. SPM analogues, receptor agonists, stable nanoparticles, and enzyme-directed strategies could enhance resolution without broadly suppressing immunity. sEH inhibitors may prolong anti-inflammatory epoxy-fatty acids, while selective modulation of cyclooxygenase or lipoxygenase branches could reshape oxylipin profiles. This therapeutic class remains experimental in IBD. The same enzymatic pathway can generate mediators with opposing actions, and systemic modulation may affect vascular tone, pain, angiogenesis, or host defence. Pharmacokinetic stability, receptor specificity, tissue delivery, and interaction with standard IBD therapies must be established before efficacy trials.

### 5.3 Redox and ferroptosis-directed approaches

The GPX4 axis suggests several interventions, including lipid-peroxide scavengers, iron modulation, selenium-dependent antioxidant support, ACSL4 inhibition, and ferroptosis suppressors. Current evidence is insufficient for clinical use. Broad antioxidant strategies can disrupt physiological redox signalling, while iron restriction could worsen a common IBD deficiency. Ferric preparations may also differ in luminal reactivity. A more defensible strategy is biomarker-led intervention. Patients with ileal CD, reduced epithelial GPX4 activity, elevated lipid-peroxidation products, or a defined stress signature might represent a testable subgroup. Any trial should monitor iron status, nutritional adequacy, infection, and mucosal healing rather than symptoms alone.

### 5.4 Microbiome-directed combinations

PUFAs, fermentable fibres, and selected microbes may interact through SCFA production, bile-acid metabolism, and lipid transformation. However, a combination product cannot be justified by adding separate pre-clinical effects. Human randomized trials should compare PUFA alone, microbiome intervention alone, combination therapy, and matched placebo when feasible. Baseline microbiome configuration should be evaluated as a prespecified effect modifier.

### 5.5 Precision nutrition

Interindividual response to PUFA exposure is biologically plausible. FADS1 genotype modifies plasma fatty-acid and metabolic responses to LA- and ALA-rich diets (Lankinen et al., 2021). Precision-nutrition frameworks for IBD therefore propose integrating diet, microbiome, clinical phenotype, and host molecular data (Sasson et al., 2021). Current personalized-diet evidence remains conceptual and methodologically heterogeneous (Wellens et al., 2023) (Table 3). Even outside IBD, EPA/DHA dose, body weight, baseline omega-3 index, age, and sex explain substantial response variability (Flock et al., 2013). Biomarkers should be used to improve causal inference, not merely to create complex prediction models. Serum profiling has shown lower concentrations of several n-3 and n-6 PUFAs in active IBD (Jiang et al., 2023). Such depletion may indicate intake, absorption, utilization, or inflammatory consumption. Serial measurements linked to diet, stool metabolomics, tissue oxylipins, and clinical activity are needed to establish directionality.

**TABLE 3:** Translational strategies, experimental model, target and challenge.

Strategy	Target	Model	Challenge
Balanced whole-diet pattern	Food matrix, fibre, microbial metabolism, nutrient adequacy	RCTs and cohorts	Cannot isolate the PUFA contribution
EPA/DHA supplement	Membrane composition and lipid mediators	RCTs	Dose, form, oxidation, timing, and responder heterogeneity
SPM analogue or delivery system	Active inflammation resolution and epithelial repair	Cell and animal	Instability, receptor selectivity, tissue exposure
sEH inhibitor	Preservation of epoxy-fatty acids	Pre-clinical colitis models	Systemic effects and no established IBD efficacy
Redox/ferroptosis modulation	GPX4, ACSL4, iron, and phospholipid peroxides	Genetic and pharmacological models	Cell-specific effects and nutritional safety
PUFA plus prebiotic/probiotic	SCFAs, bile acids, barrier and immune crosstalk	Mostly pre-clinical; limited non-IBD trials	Strain, substrate, and formulation heterogeneity
Precision nutrition	Diet, FADS genotype, redox state, microbiome, disease phenotype	Frameworks and early biomarker studies	Prediction without causal validation

## 6 Priorities for future research

### 6.1 Resolve host-microbe-lipid dynamics

The main bottleneck is the absence of longitudinal human data that simultaneously resolve dietary exposure, host lipid metabolism, microbial function, and tissue inflammation. Prospective cohorts should combine weighed or digitally assisted dietary assessment with plasma and erythrocyte fatty acids, targeted lipidomics, shotgun metagenomics, metabolomics, and validated disease-activity measures. Stable-isotope tracers could distinguish host conversion from microbial transformation. Repeated sampling before, during, and after flares would help identify whether lipid signatures precede or follow inflammation.

### 6.2 Develop biomarker-stratified nutrition trials

Uniform supplementation is unlikely to address IBD heterogeneity. A 6-month randomized trial provides a clinical starting point for biomarker-led intervention. In 60 patients with quiescent UC, faecal calprotectin ≥150 μg/g, and unchanged maintenance therapy for at least 3 months, oral EPA in free-fatty-acid form (EPA-FFA; 2 g/day) was compared with a capric/caprylic-acid triglyceride placebo. A reduction in faecal calprotectin of at least 100 μg/g occurred in 63.3% of EPA-treated patients versus 13.3% of controls, while clinical remission was maintained in 76.7% versus 50.0% (Scaioli et al., 2018). These findings support further testing in patients with residual inflammation despite symptom control; they do not establish efficacy across unselected IBD populations. Separate studies provide evidence for the other panel components. In an uncontrolled 90-day study, 19 patients with quiescent UC and elevated faecal calprotectin received oral EPA-FFA at 2 g/day. Mean erythrocyte EPA increased from 0.26% to 2.51% of total fatty acids, alongside a fall in mean faecal calprotectin from 230 to 87.7 μg/g (Prossomariti et al., 2017). This demonstrates measurable EPA incorporation, although the uncontrolled design limits causal inference. For a defined oxylipin ratio, a 2-week study in 10 patients with active CD receiving parenteral nutrition found that a fish-oil-containing lipid emulsion providing 0.6 g EPA/day increased the LTB5/LTB4 ratio generated by activated peripheral polymorphonuclear leukocytes (Ikehata et al.,1992). However this *ex vivo* ratio should not be equated with a circulating plasma ratio or assumed to behave identically during oral supplementation in UC. Together, these findings suggest that future intervention studies could combine erythrocyte PUFA profiles, faecal calprotectin and the *ex vivo* leukocyte LTB5/LTB4 ratio to assess fatty-acid status, intestinal inflammation and lipid-mediator responses, respectively. Evaluating these measures alongside clinical outcomes could help determine whether they identify patients with different responses to PUFA supplementation and inform biomarker-based stratification in subsequent trials. However, this combined panel has not been validated, and the LTB5/LTB4 ratio remains an exploratory measure whose clinical relevance requires confirmation.

### 6.3 Define generalizability beyond IBD

PUFA pathways are relevant to colorectal neoplasia, infectious enteritis, intestinal failure, radiation injury, and obesity-associated barrier dysfunction. However, shared inflammatory terminology does not imply a shared therapeutic response. Priority should be given to diseases in which altered oxylipins, epithelial lipid peroxidation, or failed resolution are demonstrated in human tissue. Each indication requires disease-specific dose, safety, and mechanistic validation.

### 6.4 Standardize clinical translation

Future trials must standardize chemical form, EPA/DHA ratio, daily dose, treatment duration, oxidation quality, food matrix, and timing relative to active disease or remission. Multicentre recruitment is essential to capture geographic dietary and microbiome variation. Pre-specified reporting should include baseline nutrition, adherence biomarkers, concomitant drugs, and safety. These measures are necessary to determine whether PUFA-directed interventions provide clinically meaningful benefit beyond contemporary IBD therapy.

## 7 Conclusion

PUFAs have bidirectional and context-dependent effects on intestinal inflammation. Outcome is determined not only by n-3 or n-6 class, but also by dose, molecular form, redox capacity, microbial metabolism, barrier state, immune phenotype, and disease stage. The microbiota-barrier-immunity axis provides the organizing network, while oxylipins, SPMs, and the GPX4-lipid peroxidation pathway are key mechanistic nodes.

The proposed pre-clinical concept of PUFA-induced metabolic enteritis is supported primarily by susceptible pre-clinical models and selected translational observations. It should not be presented as a clinical synonym for human IBD. Food-based dietary patterns, lipid-mediator pharmacology, ferroptosis-directed strategies, and microbiome combinations show mechanistic or pre-clinical potential, yet omega-3 supplementation has not consistently improved IBD outcomes in randomized trials. Longitudinal multi-omics studies and biomarker-stratified, multicentre trials are required to convert this mechanistic framework into safe and reproducible clinical nutrition.

## References

[B1] Adolph T. E. Meyer M. Schwärzler J. Mayr L. Grabherr F. Tilg H. (2022). The metabolic nature of inflammatory bowel diseases. Nat. Rev. Gastroenterol. Hepatol. 19 (12), 753–767. 10.1038/s41575-022-00658-y 35906289

[B2] Ajabnoor S. M. Thorpe G. Abdelhamid A. Hooper L. (2021). Long-term effects of increasing omega-3, omega-6 and total polyunsaturated fats on inflammatory bowel disease and markers of inflammation: a systematic review and meta-analysis of randomized controlled trials. Eur. J. Nutr. 60 (5), 2293–2316. 10.1007/s00394-020-02413-y 33084958

[B3] Albuquerque-Souza E. Dalli J. (2025). Specialized pro-resolving lipid mediators in gut immunophysiology: from dietary precursors to inflammation resolution. Curr. Opin. Clin. Nutr. Metab. Care 28 (2), 96–103. 10.1097/mco.0000000000001103 39819646

[B4] Ananthakrishnan A. N. Khalili H. Konijeti G. G. Higuchi L. M. de Silva P. Fuchs C. S. (2014). Long-term intake of dietary fat and risk of ulcerative colitis and Crohn's disease. Gut 63 (5), 776–784. 10.1136/gutjnl-2013-305304 23828881 PMC3915038

[B5] Anneberg O. M. Petersen I. S. B. Jess T. De Freitas M. B. Jalili M. (2025). The dietary inflammatory potential and its role in the risk and progression of inflammatory bowel disease: a systematic review. Clin. Nutr. 47, 146–156. 10.1016/j.clnu.2025.02.019 40022954

[B6] Ben-Mustapha Y. Rekik R. Ben-Fradj M. K. Serghini M. Sanhaji H. Ben-Ahmed M. (2024). Abnormal expression of oxylipins and related synthesizing/signaling pathways in inflammatory bowel diseases. Prostagl. Leukot. Essent. Fat. Acids 202, 102628. 10.1016/j.plefa.2024.102628 38991597

[B7] Bischoff S. C. Bager P. Escher J. Forbes A. Hébuterne X. Hvas C. L. (2023). ESPEN guideline on clinical nutrition in inflammatory bowel disease. Clin. Nutr. 42 (3), 352–379. 10.1016/j.clnu.2022.12.004 36739756

[B8] Cabré E. Mañosa M. Gassull M. A. (2012). Omega-3 fatty acids and inflammatory bowel diseases - a systematic review. Br. J. Nutr. 107 (Suppl. 2), S240–S252. 10.1017/S0007114512001626 22591898

[B9] Calder P. C. (2020). n-3 PUFA and inflammation: from membrane to nucleus and from bench to bedside. Proc. Nutr. Soc. 22, 1–13. 10.1017/S0029665120007077 32624016

[B10] Christie W. W. Harwood J. L. (2020). Oxidation of polyunsaturated fatty acids to produce lipid mediators. Essays Biochem. 64 (3), 401–421. 10.1042/EBC20190082 32618335 PMC7517362

[B11] Chulenbayeva L. Jarmukhanov Z. Kaliyekova K. Kozhakhmetov S. Kushugulova A. (2025). Quantitative alterations in short-chain fatty acids in inflammatory bowel disease: a systematic review and meta-analysis. Biomolecules 15 (7), 1017. 10.3390/biom15071017 40723889 PMC12292850

[B12] Diab J. Al-Mahdi R. Gouveia-Figueira S. Hansen T. Jensen E. Goll R. (2019). A quantitative analysis of colonic mucosal oxylipins and endocannabinoids in treatment-naïve and deep remission ulcerative colitis patients and the potential link with cytokine gene expression. Inflamm. Bowel Dis. 25 (3), 490–497. 10.1093/ibd/izy349 30476077 PMC6383859

[B13] Doll S. Proneth B. Tyurina Y. Y. Panzilius E. Kobayashi S. Ingold I. (2017). ACSL4 dictates ferroptosis sensitivity by shaping cellular lipid composition. Nat. Chem. Biol. 13 (1), 91–98. 10.1038/nchembio.2239 27842070 PMC5610546

[B14] Durkin L. A. Childs C. E. Calder P. C. (2021). Omega-3 polyunsaturated fatty acids and the intestinal Epithelium-A review. Foods 10 (1), 199. 10.3390/foods10010199 PMC7835870 33478161

[B15] Escuder-Rodríguez J. J. Liang D. Jiang X. Sinicrope F. A. (2024). Ferroptosis: biology and role in gastrointestinal disease. Gastroenterology 167 (2), 231–249. 10.1053/j.gastro.2024.01.051 38431204 PMC11193643

[B16] Feagan B. G. Sandborn W. J. Mittmann U. Bar-Meir S. D'Haens G. Bradette M. (2008). Omega-3 free fatty acids for the maintenance of remission in Crohn disease: the EPIC randomized controlled trials. JAMA 299 (14), 1690–1697. 10.1001/jama.299.14.1690 18398081

[B17] Flock M. R. Skulas-Ray A. C. Harris W. S. Etherton T. D. Fleming J. A. Kris-Etherton P. M. (2013). Determinants of erythrocyte omega-3 fatty acid content in response to fish oil supplementation: a dose-response randomized controlled trial. J. Am. Heart Assoc. 2 (6), e000513. 10.1161/JAHA.113.000513 24252845 PMC3886744

[B18] Franzosa E. A. Sirota-Madi A. Avila-Pacheco J. Fornelos N. Haiser H. J. Reinker S. (2019). Gut microbiome structure and metabolic activity in inflammatory bowel disease. Nat. Microbiol. 4 (2), 293–305. 10.1038/s41564-018-0306-4 30531976 PMC6342642

[B19] Gobbetti T. Dalli J. Colas R. A. Federici Canova D. Aursnes M. Bonnet D. (2017). Protectin D1(n-3 DPA) and resolvin D5(n-3 DPA) are effectors of intestinal protection. Proc. Natl. Acad. Sci. U. S. A. 114 (15), 3963–3968. 10.1073/pnas.1617290114 28356517 PMC5393238

[B20] Godny L. Elial-Fatal S. Arrouasse J. Sharar Fischler T. Reshef L. Kutukov Y. (2025). Mechanistic implications of the mediterranean diet in patients with newly diagnosed Crohn's disease: multiomic results from a prospective cohort. Gastroenterology 168 (5), 952–964.e2. 10.1053/j.gastro.2024.12.031 39814239

[B21] Gros B. Kaplan G. G. (2023). Ulcerative colitis in adults: a review. JAMA 330 (10), 951–965. 10.1001/jama.2023.15389 37698559

[B22] Haskey N. Estaki M. Ye J. Shim R. K. Singh S. Dieleman L. A. (2023). A mediterranean diet pattern improves intestinal inflammation concomitant with reshaping of the bacteriome in ulcerative colitis: a randomised controlled trial. J. Crohns Colitis 17 (10), 1569–1578. 10.1093/ecco-jcc/jjad073 37095601 PMC10637046

[B23] IBD in EPIC Study Investigators Tjonneland A. Overvad K. Bergmann M. M. Nagel G. Linseisen J. (2009). Linoleic acid, a dietary n-6 polyunsaturated fatty acid, and the aetiology of ulcerative colitis: a nested case-control study within a European prospective cohort study. Gut 58 (12), 1606–1611. 10.1136/gut.2008.169078 19628674

[B24] Ikehata A. Hiwatashi N. Kinouchi Y. Yamazaki H. Kumagai Y. Ito K. (1992). Effect of intravenously infused eicosapentaenoic acid on the leukotriene generation in patients with active Crohn's disease. Am. J. Clin. Nutr. 56 (5), 938–942. 10.1093/ajcn/56.5.938 1329484

[B25] Jiang J. Chen L. Sun R. Yu T. Jiang S. Chen H. (2023). Characterization of serum polyunsaturated fatty acid profile in patients with inflammatory bowel disease. Ther. Adv. Chronic Dis. 14, 20406223231156826. 10.1177/20406223231156826 37008284 PMC10052691

[B26] Jones R. D. Liao J. Tong X. Xu D. Sun L. Li H. (2019). Epoxy-Oxylipins and soluble epoxide hydrolase metabolic pathway as targets for NSAID-induced gastroenteropathy and inflammation-associated carcinogenesis. Front. Pharmacol. 10, 731. 10.3389/fphar.2019.00731 31293429 PMC6603234

[B27] Lankinen M. A. de Mello V. D. Meuronen T. Sallinen T. Ågren J. Virtanen K. A. (2021). The FADS1 genotype modifies metabolic responses to the linoleic acid and alpha-linolenic acid containing plant oils-genotype based randomized trial FADSDIET2. Mol. Nutr. Food Res. 65 (7), e2001004. 10.1002/mnfr.202001004 33548080

[B28] Lewis J. D. Sandler R. S. Brotherton C. Brensinger C. Li H. Kappelman M. D. (2021). A randomized trial comparing the specific carbohydrate diet to a mediterranean diet in adults with Crohn's disease. Gastroenterology 161 (3), 837–852.e9. 10.1053/j.gastro.2021.05.047 PMC8396394 34052278

[B29] Liu F. Smith A. D. Solano-Aguilar G. Wang T. T. Y. Pham Q. Beshah E. (2020). Mechanistic insights into the attenuation of intestinal inflammation and modulation of the gut microbiome by krill oil using *in vitro* and *in vivo* models. Microbiome 8 (1), 83. 10.1186/s40168-020-00843-8 32498703 PMC7273679

[B30] Lloyd-Price J. Arze C. Ananthakrishnan A. N. Schirmer M. Avila-Pacheco J. Poon T. W. (2019). Multi-omics of the gut microbial ecosystem in inflammatory bowel diseases. Nature 569 (7758), 655–662. 10.1038/s41586-019-1237-9 31142855 PMC6650278

[B31] Lo C. H. Lochhead P. Khalili H. Song M. Tabung F. K. Burke K. E. (2020). Dietary inflammatory potential and risk of Crohn's disease and ulcerative colitis. Gastroenterology 159 (3), 873–883.e1. 10.1053/j.gastro.2020.05.011 32389666 PMC7502466

[B32] Marsh A. Chachay V. Banks M. Okano S. Hartel G. Radford-Smith G. (2024). A pilot randomized controlled trial investigating the effects of an anti-inflammatory dietary pattern on disease activity, symptoms and microbiota profile in adults with inflammatory bowel disease. Eur. J. Clin. Nutr. 78 (12), 1072–1081. 10.1038/s41430-024-01487-9 39122803 PMC11611722

[B33] Massironi S. Viganò C. Palermo A. Pirola L. Mulinacci G. Allocca M. (2023). Inflammation and malnutrition in inflammatory bowel disease. Lancet Gastroenterol. Hepatol. 8 (6), 579–590. 10.1016/s2468-1253(23)00011-0 36933563

[B34] Mayr L. Grabherr F. Schwärzler J. Reitmeier I. Sommer F. Gehmacher T. (2020). Dietary lipids fuel GPX4-restricted enteritis resembling Crohn's disease. Nat. Commun. 11 (1), 1775. 10.1038/s41467-020-15646-6 32286299 PMC7156516

[B35] Meyer A. Agrawal M. Savin-Shalom E. Wong E. C. L. Levinson C. Gold S. (2025). Impact of diet on inflammatory bowel disease risk: systematic review, meta-analyses and implications for prevention. EClinicalMedicine 86, 103353. 10.1016/j.eclinm.2025.103353 PMC12281061 40697960

[B36] Miranda J. Brazil J. C. Morris A. H. Parkos C. A. Quiros M. Nusrat A. (2023). Maresin-2 promotes mucosal repair and has therapeutic potential when encapsulated in thermostable nanoparticles. Proc. Natl. Acad. Sci. U. S. A. 120 (4), e2218162120. 10.1073/pnas.2218162120 PMC9942869 36669099

[B37] Mozaffari H. Daneshzad E. Larijani B. Bellissimo N. Azadbakht L. (2020). Dietary intake of fish, n-3 polyunsaturated fatty acids, and risk of inflammatory bowel disease: a systematic review and meta-analysis of observational studies. Eur. J. Nutr. 59 (1), 1–17. 10.1007/s00394-019-01901-0 30680455

[B38] Nieva C. Pryor J. Williams G. M. Hoedt E. C. Burns G. L. Eslick G. D. (2024). The impact of dietary interventions on the microbiota in inflammatory bowel disease: a systematic review. J. Crohns Colitis 18 (6), 920–942. 10.1093/ecco-jcc/jjad204 PMC11147801 38102104

[B39] Prossomariti A. Scaioli E. Piazzi G. Fazio C. Bellanova M. Biagi E. (2017). Short-term treatment with eicosapentaenoic acid improves inflammation and affects colonic differentiation markers and microbiota in patients with ulcerative colitis. Sci. Rep. 7 (1), 7458. 10.1038/s41598-017-07992-1 28785079 PMC5547132

[B40] Ren Y. Wang T. Yin J. (2024). The role of soluble epoxide hydrolase in the intestine. Cell Biol. Int. 48 (11), 1612–1620. 10.1002/cbin.12232 39164961

[B41] Roda G. Chien Ng S. Kotze P. G. Argollo M. Panaccione R. Spinelli A. (2020). Crohn's disease. Nat. Rev. Dis. Prim. 6 (1), 22. 10.1038/s41572-020-0156-2 32242028

[B42] Ruan G. Sun Y. Yu Z. Bai X. Yang H. Qian J. (2025). Global, regional, and national burden of inflammatory bowel disease from 1990 to 2021: findings from the global burden of disease 2021. Gastroenterol. Rep. (Oxf) 13, goaf082. 10.1093/gastro/goaf082 40979945 PMC12448184

[B43] Sasson A. N. Ingram R. J. M. Zhang Z. Taylor L. M. Ananthakrishnan A. N. Kaplan G. G. (2021). The role of precision nutrition in the modulation of microbial composition and function in people with inflammatory bowel disease. Lancet Gastroenterol. Hepatol. 6 (9), 754–769. 10.1016/s2468-1253(21)00097-2 34270915

[B44] Scaioli E. Sartini A. Bellanova M. Campieri M. Festi D. Bazzoli F. (2018). Eicosapentaenoic acid reduces fecal levels of Calprotectin and prevents relapse in patients with ulcerative colitis. Clin. Gastroenterol. Hepatol. 16 (8), 1268–1275.e2. 10.1016/j.cgh.2018.01.036 29391271

[B45] Schwärzler J. Mayr L. Vich Vila A. Grabherr F. Niederreiter L. Philipp M. (2022). PUFA-induced metabolic enteritis as a fuel for Crohn's disease. Gastroenterology 162 (6), 1690–1704. 10.1053/j.gastro.2022.01.004 35031299

[B46] Seethaler B. Lehnert K. Yahiaoui-Doktor M. Basrai M. Vetter W. Kiechle M. (2023). Omega-3 polyunsaturated fatty acids improve intestinal barrier integrity-albeit to a lesser degree than short-chain fatty acids: an exploratory analysis of the randomized controlled LIBRE trial. Eur. J. Nutr. 62 (7), 2779–2791. 10.1007/s00394-023-03172-2 37318580 PMC10468946

[B47] Storniolo C. E. Pequera M. Vilariño A. Moreno J. J. (2022). Specialized pro-resolvin mediators induce cell growth and improve wound repair in intestinal epithelial Caco-2 cell cultures. Prostagl. Leukot. Essent. Fat. Acids 187, 102520. 10.1016/j.plefa.2022.102520 36427427

[B48] Vijay A. Astbury S. Le Roy C. Spector T. D. Valdes A. M. (2021). The prebiotic effects of omega-3 fatty acid supplementation: a six-week randomised intervention trial. Gut Microbes 13 (1), 1–11. 10.1080/19490976.2020.1863133 33382352 PMC7781624

[B49] Wang F. Lin X. Zhao Q. Li J. (2017). Fat intake and risk of ulcerative colitis: systematic review and dose-response meta-analysis of epidemiological studies. J. Gastroenterol. Hepatol. 32 (1), 19–27. 10.1111/jgh.13416 27097307

[B50] Watson H. Mitra S. Croden F. C. Taylor M. Wood H. M. Perry S. L. (2018). A randomised trial of the effect of omega-3 polyunsaturated fatty acid supplements on the human intestinal microbiota. Gut 67 (11), 1974–1983. 10.1136/gutjnl-2017-314968 28951525

[B51] Wawrzyniak P. Noureddine N. Wawrzyniak M. Lucchinetti E. Krämer S. D. Rogler G. (2021). Nutritional lipids and mucosal inflammation. Mol. Nutr. Food Res. 65 (5), e1901269. 10.1002/mnfr.201901269 32780927

[B52] Wellens J. Vissers E. Matthys C. Vermeire S. Sabino J. (2023). Personalized dietary regimens for inflammatory bowel disease: current knowledge and future perspectives. Pharmgenomics Pers. Med. 16, 15–27. 10.2147/pgpm.s359365 36660362 PMC9842524

[B53] Yan J. Zeng Y. Guan Z. Li Z. Luo S. Niu J. (2024). Inherent preference for polyunsaturated fatty acids instigates ferroptosis of treg cells that aggravates high-fat-diet-related colitis. Cell Rep. 43 (8), 114636. 10.1016/j.celrep.2024.114636 39154340

[B54] Yanai H. Levine A. Hirsch A. Boneh R. S. Kopylov U. Eran H. B. (2022). The Crohn's disease exclusion diet for induction and maintenance of remission in adults with mild-to-moderate Crohn's disease (CDED-AD): an open-label, pilot, randomised trial. Lancet Gastroenterol. Hepatol. 7 (1), 49–59. 10.1016/s2468-1253(21)00299-5 34739863

[B55] Zhang W. Li H. Dong H. Liao J. Hammock B. D. Yang G. Y. (2013). Soluble epoxide hydrolase deficiency inhibits dextran sulfate sodium-induced colitis and carcinogenesis in mice. Anticancer Res. 33 (12), 5261–5271. 24324059 PMC4076172

